# Optimization and Chemical Characterization of Extracts Obtained from *Ferula persica* var. *latisecta* Aerial Parts and Roots and Their Neuroprotective Evaluation

**DOI:** 10.3390/nu16234210

**Published:** 2024-12-05

**Authors:** Pouya Mohammadnezhad, Alberto Valdés, Alejandro Cifuentes

**Affiliations:** Foodomics Laboratory, Instituto de Investigacion en Ciencias de la Alimentacion (CIAL), Consejo Superior de Investigaciones Científicas-Universidad Autónoma de Madrid (CSIC-UAM), Nicolas Cabrera 9, 28049 Madrid, Spain; p.mohammadnezhad@cial.uam-csic.es (P.M.); a.cifuentes@csic.es (A.C.)

**Keywords:** green extraction, HPLC/GC-MS, molecular docking, neuroprotection, pressurized liquid extraction, sesquiterpenoids

## Abstract

Background/Objectives: The genus *Ferula* has been traditionally used for the treatment of various illnesses, but the potential of *Ferula persica* var. *latisecta* against different Alzheimer’s disease (AD) hallmarks has never been achieved. Methods: In this work, a pressurized liquid extraction (PLE) method was optimized to extract *F. persica* L. aerial parts and roots. Four different solvents (water, ethanol, ethyl acetate (EtAc), and cyclopentyl methyl ether (CPME)) were first tested, and the extraction yield, total phenolic content, reactive oxygen species scavenging capacity, and acetylcholinesterase (AChE) inhibition activity were evaluated. Results: The results indicated that EtAc and CPME were the best solvents to be used, with the results obtained from the aerial parts being better than those obtained from the root samples. Thereafter, the PLE method was further optimized by combining these solvents in different percentages (100% EtAc, 100% CPME, and 50:50% (*v*/*v*) EtAc:CPME) and temperatures (50, 115, and 180 °C). Response surface methodology was then applied to analyze the data, and two optimum extraction conditions were obtained: EtAc:CPME (79:21%) at 180 °C for the aerial parts and 100% CPME at 180 °C for the roots. At these conditions, the total flavonoid content (TFC) and the inhibitory capacities against butyrylcholinesterase (BChE) and lipoxygenase (LOX) enzymes were also evaluated, indicating that the aerial part extracts had higher TFC and LOX inhibitory capacity than the root extracts but lower activity against BChE. The comprehensive LC/GC-MS chemical characterization allowed for the tentative identification of 222 compounds belonging to 66 chemical subclasses, the abundancies of which widely varied depending on the matrix and the extraction conditions used. Conclusions: The results obtained together with the application of advanced statistical analysis and molecular docking simulations suggested several sesquiterpenoids, such as selina-3,7(11)-diene, guaiol acetate, α-cyperone, and farnesyl acetate, as the molecules responsible of the in vitro results observed, with good neuroprotective potential against AD.

## 1. Introduction

Plants have a rich historical background as therapeutic agents in the treatment of human diseases and they have been used as a source of medicine throughout the ages. In traditional medicine, numerous plants and specific plant organs (leaves, stems, roots, flowers, fruits, or seeds) are appreciated for their medicinal properties, some of them related to central neuronal system (CNS) diseases [1]. In the last decades, there has been growing interest in bioactive compounds obtained from natural sources and researchers have significantly contributed to the identification of various natural compounds that exhibit antioxidant, anti-inflammatory, and anti-cholinesterase (ChE) activities [2,3,4].

The genus *Ferula* belongs to the family Apiaceae and it comprises approximately 130 species distributed across the Mediterranean area and Central Asia [5]. Different species from this genus have been utilized for their therapeutic properties for many centuries [5]. It has been reported that the genus *Ferula* possesses a diverse array of pharmacological properties, including chemopreventive effects against cancer, anti-inflammatory and lipoxygenase inhibitory activities, reversal of multidrug resistance, and antimicrobial activity [6,7]. *Ferula persica* is a well-known species of the genus *Ferula*, and the main varieties in Iran are *persica* and *latisecta* [7]. These varieties have diverse bioactive properties and uses. The aerial parts of *Ferula latisecta* were traditionally used to alleviate stomachache in infants, while the resin of *Ferula badrakema* and the root of *Ferula diversivittata* were utilized as anticonvulsants, tonics, anti-hysteric agents, and decongestants [8]. Additionally, the root of *Ferula persica* served as a laxative, carminative, and anti-hysteric agent, and it was used for treating lumbago, diabetes, rheumatism, and backache [8,9]. *Ferula persica* also exhibits various effects on the nervous system, including analgesic, anti-cramp, and antispasmodic activities, particularly in ileum contraction [10,11]. Furthermore, specific reports have highlighted the traditional use of specific *Ferula* species in the treatment of neurological disorders or for enhancing memory [12], but the beneficial properties of this plant have scarcely been investigated against Alzheimer’s disease (AD) hallmarks.

AD is characterized by progressive neuron loss in diverse regions of the brain [13], it is associated with cognitive impairment and behavioral disturbances [12,14,15], and it comprises 60–70% of reported dementia cases [16]. The deposition of ß-amyloid peptides and the accumulation of neurofibrillary tangles, together with the activities of acetylcholinesterase (AChE), butyrylcholinesterase (BChE), and lipoxygenases (LOX) enzymes, have demonstrated crucial roles in neurological disorders, regulating the cholinergic system and participating in neuroinflammatory responses [17]. Moreover, neurons are susceptible to oxidative stress caused by reactive oxygen species (ROS) and reactive nitrogen species, leading to mitochondrial alterations, neuronal cell death, or glutamate excitotoxicity [18,19]. However, despite having all of this knowledge, the multifactorial nature of this disorder coupled with its complex interactions confers significant challenges to drug development. Moreover, the limited efficacy of traditional medications and the high failure rates in drug development due to insufficient efficacy or adverse effects has enhanced the search for new alternatives [4]. In this regard, nutraceuticals and bioactive compounds have emerged as promising therapeutic approaches that could prevent or retard AD occurrence owing to their multiple neuroprotective mechanisms [20,21].

Among the available natural sources, the genus *Ferula* is well documented as a rich source of biologically active compounds, especially sesquiterpene coumarins [5,22,23]. Moreover, a variety of chemical constituents, such as polysulfides and volatile compounds, have been isolated from *F. persica* [7]. To extract these health-beneficial compounds, conventional techniques such as agitation, maceration, and Soxhlet extraction are commonly used, but they are known to require prolonged extraction times [24,25]. By contrast, the utilization of advanced environmentally friendly extraction techniques, such as compressed fluids (e.g., pressurized liquid extraction (PLE) and supercritical fluid extraction (SFE)), offers several advantages, such as reducing organic solvent volume and extraction times, as well as enhancing the extraction yield of bioactive compounds by increasing the mass transfer rate between the solvent and the biomass [26]. For instance, PLE has proven its suitability for extracting bioactive compounds from plants, including phenolic and terpenoid compounds, and these compounds have been identified as potential neuroprotective molecules owing to their anti-cholinesterase, antioxidant, and anti-inflammatory capacities [27,28,29].

Based on the above information, we hypothesize that the aerial parts and roots of *F. persica* L. could be a good source of compounds with multifaceted potential against different Alzheimer’s disease (AD) hallmarks and can be obtained using compressed fluids. Therefore, the present study aims to optimize the extraction process based on different response variables (such as the total phenolic/flavonoid content and the evaluation of diverse in vitro neuroprotective mechanisms related to AD), to perform a comprehensive chemical characterization of the extracts using HPLC-MS/MS and GC-MS to identify the bioactive compounds that could be responsible for the observed in vitro results, and to apply advanced statistical analysis and molecular docking simulations to select and evaluate the affinity of the most promising compounds for the active sites of different enzymes related to AD.

## 2. Materials and Methods

### 2.1. Chemicals and Reagents

Folin–Ciocalteu reagent was procured from Merck (Darmstadt, Germany). Trizma hydrochloride (Tris-HCl), acetylcholinesterase (AChE), butyrylcholinesterase (BChE), naphthylethylene diamine dihydrochloride, sulfanilamide, acetylthiocholine iodide, linoleic acid, aluminum chloride, phosphoric acid, sodium carbonate, potassium phosphate, monopotassium phosphate, sodium nitroprusside dehydrate, fluorescein, gallic acid, quercetin, galantamine hydrobromide, ascorbic acid, and a 96-well acceptor plate (Catalog no MATRNPS50) were purchased from Sigma-Aldrich (Madrid, Spain). Lipoxygenases from glycine max (soybean), 2,2-azobis(2-amidinopropane) dihydrochloride (AAPH), and 4-(aminosulfonyl)-7-fluoro-2,1,3-benzoxadiazole (ABD-F) were obtained from TCI Chemicals (Tokyo, Japan). LC-MS-grade acetonitrile (ACN), LC-MS-grade methanol, cyclopentyl methyl ether (CPME), ethyl acetate (EtAc), and ethanol (EtOH) were obtained from VWR Chemicals (Barcelona, Spain), whereas Milli-Q water was obtained from a Millipore system (Billerica, MA, USA). Formic acid was purchased from Fisher Scientific (Waltham, MA, USA).

### 2.2. Sample Preparation

The entire *Ferula persica* L. plant, including the leaves, roots, twigs, and stems, was collected from the mountains near to the city of Shahmirzad, located in the Semnan province of Iran, at approximately 35°46′09″ N latitude and 53°20′10″ E longitude. The different parts of the plant were air-dried at room temperature for 4 days until they reached a constant weight. After drying, the samples were stored at −18 °C for eight weeks before analysis. For experiments, the aerial parts (leaves, twigs, and flowers) and the roots were homogenized independently and sieved with a 250–500 μm size mesh.

### 2.3. Supercritical Fluid Extraction (SFE) of Ferula persica L.

Supercritical fluid extraction (SFE) of *F. persica* aerial parts and roots was performed using the same homemade compressed fluid extraction system as in [30]. CO_2_ from a cylinder was cooled and compressed by a CO_2_ pump from Jasco (Tokyo, Japan). Then, preheated CO_2_ was directed toward the oven, where the extraction cell containing the sample was placed. The flow rate was adjusted to 4 mL/min, and the pressure (300 bar) was regulated by manipulating the opening of two needle valves. The extraction cell, filled with a mixture of 1.5 g of sample and 3 g of sand positioned between two layers of glass wool, underwent extraction for 120 min at a temperature of 40 °C. At the end of the process, the extract recovery system was cooled through CO_2_ expansion and shielded from light using aluminum foil. These experiments were conducted in triplicate.

### 2.4. Pressurized Liquid Extraction (PLE) of Ferula persica L.

Pressurized liquid extraction (PLE) was performed using an accelerated solvent extractor (ASE 200, Dionex, Sunnyvale, CA, USA), equipped with a solvent controller. In this process, 1 g of *F. persica* aerials parts or roots was mixed with 2 g of sand and placed into an 11 mL extraction cell. Extraction was then carried out using the following parameters: time, 20 min; pressure, 10 MPa, heat-up time, 5 min; static extraction time, 20 min; flush volume, 60%; purge, N_2_ for 90 s; number of cycles, 1. The purged sample extracts were collected in collection vials by compressed gas. Finally, the extracts were protected from light, dried under N_2_ stream, and stored at −20 °C until further use. Initially, four different solvents were employed for the extraction: H_2_O, EtOH, EtAc, and CPME. Among these solvents, EtAc and CPME provided the best results (see Section 3.1) for the aerial parts and roots. Consequently, the PLE method was further optimized by combining these solvents in different percentages (100% EtAc, 100% CPME, and 50:50% (*v*/*v*) EtAc:CPME) and different temperatures (50, 115, and 180 °C) following a central composite design (CCD) and including 3 replicates at the central point. After the extraction, the extracts were dried under nitrogen flow, and the extraction yield, TPC, ROS scavenging capacity, and AChE inhibitory activity were evaluated as response variables utilizing response surface methodology (RSM). The analysis was conducted using Statgraphics Centurion XVI software (v.16.1.11) (StatPoint Technologies, Inc., Warrenton, VA, USA).

### 2.5. Extraction Yield, Total Phenolic Content and Total Flavonoid Content

The extraction yield was expressed as the percentage of the extract mass on a dry basis and the mass of the initial *F. persica* aerial parts and roots fed into the SFE or the PLE extraction cell. The TPC and TFC of the *F. persica* PLE extracts were assessed according to previously published methods [31,32]. For TPC measurement, the calibration curve was established using 0.031–2 mg gallic acid/mL in EtOH, and it was used to calculate the TPC of the *F. persica* PLE extracts expressed as milligrams of gallic acid equivalents per gram of extract (mg GA/g extract). For TFC, the results were expressed as milligrams of quercetin equivalents per gram of extract (mg QE/g extract). All measurements were performed in triplicate using a 96-well microplate reader (Synergy HT, BioTek Instruments, Winooski, VT, USA).

### 2.6. ROS Scavenging Capacity, Anti-Cholinergic and Lipoxygenase Inhibitory Activities

The ROS scavenging capacity of the *F. persica* PLE extracts was measured using the oxygen radical absorbance capacity (ORAC) assay, as previously described [33,34]. Ascorbic acid and EtOH were utilized as the reference standard and blank control solutions, respectively. The AChE and BChE inhibitory activities of the *F. persica* PLE extracts were assessed using the fluorescent enzyme kinetic method described in [34]. Galantamine hydrobromide was used as the reference inhibitor and 50% EtOH was used as blank control. Finally, the LOX inhibitory activity of the *F. persica* PLE extracts was determined as described in [35] with slight modifications [34] to adapt the protocol for a 96-well microplate reader. Quercetin was used as a reference inhibitor and 25% EtOH was used as a blank control.

### 2.7. Chemical Characterization of Ferula persica L. PLE Extract

The chemical characterization of the *F. persica* aerial part and root PLE extracts obtained using the four initial solvents (H_2_O, EtOH, EtAc, and CPME) at 115 °C and under optimum conditions (Aerial Opt and Root Opt) was performed in triplicate. Each extract was dissolved in EtOH to a final concentration of 3 mg/mL. Subsequently, the samples were vortexed for 30 s, centrifuged at 14,800 rpm for 5 min at 4 °C, and the supernatants were collected and stored at −80 °C until analysis. Aliquots of 2 μL were injected into a LC-MS/MS system consisting of a HPLC model 1290 coupled to a Q-TOF series 6540 using an Agilent Jet Stream thermal orthogonal ESI source (Agilent Technologies, Waldbronn, Germany). Compounds were separated using a ZORBAX Eclipse Plus C18 analytical column (100 mm length × 2.1 mm i.d., 1.8 μm particle size) and a C18 guard column (0.5 cm× 2.1 mm, 1.8 μm particle size), both from Agilent Technologies (Wadbronn, Germany). Milli-Q water was used as mobile phase (A), while acetonitrile (ACN) was employed mobile phase (B), and 0.1% formic acid was used as the mobile phase modifier. The column temperature was maintained at 40 °C, and the flow rate was set to 0.5 mL/min with the following gradient: 0–30% B in 7 min; 30–80% B in 2 min; 80–100% B in 2 min; 100% B for 2 min; 100–0% B in 1 min; 0% B for 3 min. The mass spectrometer was operated in ESI (+) and ESI (−) modes using the same parameters as previously described [36]. The chromatograms were analyzed using MS-DIAL v4.9 software to obtain the relative abundances (as area under the curve (AUC)) and for the tentative identification of the compounds (by comparison with MS/MS spectra from NIST, LipidBLAST, and MoNA databases). All data were manually verified.

Additionally, the analysis of extracts was performed employing an Agilent 7890B gas chromatography (GC) system coupled to an Agilent 7200 Q-TOF mass spectrometer, equipped with an electronic ionization (EI) interface. Aliquots of 1 μL of the samples were injected with a split ratio of 1:10 and separated using an Agilent Zorbax DB5-MS column (30 m × 250 μm i.d. × 0.25 μm) + 10 m DuraGuard capillary column. The programmed temperature gradient started at 60 °C (1 min), rising at 10 °C/min to 325 °C, and this temperature was held for 10 min, using a constant flow of 1 mL/min of He. The MS detector was operated in full-scan acquisition mode at a *m*/*z* scan range of 20–600 Da with an ionizing voltage of 70 eV. The temperatures of the transfer line, the quadrupole, and the ion source were set at 290, 150, and 250 °C, respectively. Mass spectra deconvolution of the chromatographic signals was performed using Agilent Mass Hunter Unknown Analysis software (v10.0), and the tentative identification of the compounds was performed using the NIST MS Search v.2.3 and Fiehn Lib databases. Retention indices (RI) were determined using the retention times of *n*-alkanes (C10–C30) that were injected after the extracts under identical chromatographic conditions. The compounds were identified by comparison of the retention indices (RI, semi-standard non-polar) with those contained in the NIST database or reported in the literature. MS-DIAL v4.9 software was used to obtain the relative abundances of the compounds (as AUC). All data were manually verified.

### 2.8. Molecular Docking

Molecular docking simulations were performed to evaluate the affinity of selected compounds for the active sites of AChE, BChE, and LOX enzymes. The current docking study used the published crystal structures of the proteins in complex with different inhibitors, including AChE-huprine W (PDB ID: 4BDT), BChE-tacrine (PDB ID: 4BDS) [37], and LOX-epigallocatechin gallate (PDB ID: 1JNQ) [38], which were retrieved from the Protein Data Bank (PDB) (http://www.rscb.org/pdb, accessed on 22 July 2024). First, Discovery Studio 2024 (Dassault Systèmes Biovia Corp, San Diego, CA, USA) was used to obtain the coordinates for the active site of each protein structure: AChE (X = −2.086682; Y = −35.174500; Z = −51.681500), BChE (X = 132.994467; Y = 116.013533; Z = 41.214400), and LOX (X = 20.503864; Y = 3.236000; Z = 19.853727). Thereafter, the 3 proteins, 19 ligands (acetylleucine, 4-methyl-5-thiazoleethanol, β-asarone, *N*-(2-phenylethyl)acetamide, farnesyl acetate, thymidine, cis,cis-linoleic acid, isofraxidin, kaempferol, α-hydroxybutyric acid, nobiletin, γ-sitosterol, guaiol acetate, selina-3,7(11)-diene, 2-hydroxypalmitic acid, stearic acid, palmitic acid, palmitoleic acid, and α-cyperone), and the standard inhibitors galantamine and quercetin were prepared according to [39] using Chimera software (v. 1.16). For the molecular docking process, a grid box with a point spacing of 0.375 Å and dimensions of 23 × 23 × 23 was built in the active site of each enzyme, and one ligand at a time was docked to each protein using AutoDock Vina (v. 1.2.5) [40]. The results were expressed as binding energies.

### 2.9. Statistical Analysis

Principal component analysis (PCA), partial least squares discriminant analysis (PLS-DA), and heatmap analysis were performed using the MetaboAnalyst 6.0 website tool (https://www.metaboanalyst.ca, accessed on 9 June 2024). Dendrogram analysis and chemical similarity enrichment (ChemRICH) analysis for multiple conditions were performed in R (v.4.2.1) using the ape package (v.5.8) and the ChemRICH package (https://chemrich.idsl.me/run-chemrich/for-multiple-conditions, accessed on 20 June 2024). *t*-tests and ANOVA were carried out using STATISTICA software (v.7.1 StatSoft, Inc., Tulsa, OK, USA), and differences between metabolites were considered significant when *p* < 0.05.

## 3. Results and Discussion

### 3.1. Green Compressed Fluids to Extract Bioactive Compounds from Ferula persica L. Aerial Parts and Roots

The extraction of bioactive compounds from *F. persica* aerial parts and roots was initially performed using two different technologies: SFE (using CO_2_ as solvent) and PLE (using H_2_O, EtOH, EtAc, and CPME). In the case of SFE, the extraction performed at 300 bar and 40 °C for 2 h provided a low extraction yield (<1%), and therefore this technology was discarded for further analyses. In the case of PLE, the extraction yield varied depending on the different solvents and matrices used (Table 1). In both the aerial parts and roots, H_2_O demonstrated the best extraction yield, followed by EtOH, and finally EtAc or CPME. With respect to the different matrices, the extraction yield was significantly higher for the aerial parts as compared to the roots when H_2_O and EtOH were used, but there were no differences when using EtAc and CPME. These results agreed with previous works demonstrating that pressurized H_2_O can increase the extraction yield due to better extraction of carbohydrates and proteins [41]. However, a higher extraction yield does not necessarily indicate better bioactive properties, and therefore the TPC, ROS scavenging capacity, and AChE inhibitory activity of all of the PLE extracts were measured. As can be observed in Table 1, all of these parameters varied depending on the different solvents and matrices used. In both matrices, better results were achieved when utilizing non-polar solvents, such as EtAc and CPME, with those obtained from the aerial parts being better than those obtained from the root samples. It must be mentioned that the ROS and AChE results are expressed as IC_50_ (μg/mL), meaning that higher activities were achieved when lower IC_50_ values were obtained. These findings suggested that the nature of the compounds contributing to the TPC, ROS, and AChE activities might be relatively non-polar (see Section 3.4). Based on these results, EtAc and CPME solvents were selected for further optimization of the PLE extraction method.

### 3.2. Optimization of the PLE Extraction Conditions for Aerial Parts and Roots

Owing to the good results obtained with EtAc and CPME, the PLE conditions were optimized using a CCD approach. With that aim, the two solvents were combined at different percentages (100% EtAc, 100% CPME, and 50:50% (*v*/*v*) EtAc:CPME) and temperatures (50, 115, and 180 °C), and the extraction yield, TPC, ROS, and AChE activities were measured and analyzed as response variables in RSM. The results obtained for the aerial parts and roots are shown in Table 2 and Table 3, respectively.

Table 2 shows that the extraction yield of the aerial part extracts substantially increased for all solvent compositions when the temperature was increased, being the highest when 100% EtAc at 180 °C was used. On the other hand, the lowest value was obtained with 50% EtAc:CPME at 50 °C. In the case of TPC, the highest values were obtained with 100% EtAc, 50% EtAc:CPME, and 100% EtAc at the highest temperature of 180 °C, while the lowest values were obtained with 100% CPME and 100% EtAc at the lowest temperature of 50 °C. In the case of the ROS and AChE assays, the IC_50_ values were decreased at 180 °C. The best results for these assays were achieved under different conditions: for ROS, the best result was obtained with 100% EtAc at 180 °C, whereas for AChE, the best value was achieved when using 50% EtAc:CPME at 180 °C. Based on these results, the optimum PLE conditions for the aerial part samples and calculated using RSM was a mixture of EtAc and CPME (79:21% (*v*/*v*)) at 180 °C (named as Aerial Opt) (Table S1).

Similarly to the aerial part samples, the maximum extraction yield of the root samples was also achieved when the temperature was increased to 180 °C (Table 3). However, the highest extraction yield of the root samples was attained when using 100% CPME at 180 °C, which was lower than the maximum obtained from the aerial part samples when using 100% EtAc at the same temperature. Under this condition of 100% CPME at 180 °C, the highest value for TPC, the best value for AChE, and the second-best value for ROS were achieved (the best ROS scavenging capacity was obtained when using 100% EtAc at 180 °C). Based on these results and using RSM, the optimum PLE condition for the root samples was 100% CPME at 180 °C (named as Root Opt) (Table S1).

### 3.3. Comparison Between Aerial Parts and Roots Under PLE Optimum Conditions

Once the PLE conditions were optimized, three independent experiments were performed under each extraction condition to experimentally confirm the predictive values obtained for TPC, ROS, and AChE. In addition, three more complementary assays were performed (TFC, BChE inhibitory capacity, and LOX inhibitory activity) to obtain a wider view of the neuroprotective and anti-inflammatory potential of the obtained extracts (Table 4).

Table 4 shows that the extraction yield for Aerial Opt was significantly higher than that obtained for Root Opt, which was somehow expected based on the results obtained in Table 2 and Table 3. In addition, the extraction yields for Aerial Opt and Root Opt were better than those predicted by the RSM model (Table S1). The TPC values also agreed with the previous results and the predicted values and showed a higher amount in the Root Opt extract compared to the Aerial Opt extract. However, the TFC value obtained for the Aerial Opt extract was significantly higher than that obtained for the Root Opt extract. This result was in line with a recently published article that indicated that the flavonoid concentration is higher in the aerial parts of plants compared to roots across most species [42], as is the case for different *Bupleurum* species belonging to the family Apiaceae [43]. In addition, the results presented in Table S2 showed that the amounts of most flavonoids (except nobiletin and hesperidin) were higher in the aerial parts than in the root samples. The TPC and TFC values also aligned with those obtained from the aerial parts of *F. persica* by maceration for 24 h with EtOH [25] but were lower than those obtained from the aerial parts and roots by maceration for 24 h with EtAc [24]. This effect might have been due to the shorter extraction time used (20 min), which can decrease the extraction yield. However, this reduction did not necessarily mean a reduction in bioactivity, as this activity depends on the selective extraction of specific compounds. Moreover, shorter extraction times combined with the absence of oxygen during the extraction and the highly controlled temperature of the PLE system can avoid the degradation and/or the occurrence of secondary reactions of valuable compounds.

In the case of ROS scavenging capacity, there were no significant differences between the Aerial Opt and Root Opt extracts. The ROS value obtained for the Root Opt extract was identical to that obtained under the same experimental conditions performed during the PLE optimization process with CPME 100% at 180 °C (Table 3); but, the results for the Aerial Opt extract were slightly lower than those predicted by the RSM model. However, both extracts had higher activity than ascorbic acid, the reference scavenger of ROS used in this study (Table 4). These results were in good agreement with a previous study that showed that *F. persica* extracts can serve as a potential antioxidant agent that can suppress free radicals and inhibit oxidation reactions [44].

In terms of ChE inhibitory capacity, the Aerial Opt extract showed a significantly higher activity against AChE compared to the Root Opt extract, whereas the activity against BChE was higher for the Root Opt extract. This is the first time that ChE inhibitory capacity has been reported for *F. persica* extracts, and the different effects observed in both enzymes may have been due to the significant differences in the TPC and TFC values and the diverse chemical composition of the extracts (see Section 3.4). In this regard, previous studies have demonstrated that various phenolic acids and flavonoid derivatives have significantly different activities against AChE and BChE enzymes [45,46].

Finally, in vitro neuro-inflammatory protection was evaluated through LOX enzymatic inhibition. The IC_50_ value for LOX inhibitory capacity was lower for the Aerial Opt extract than for the Root Opt extract. Moreover, the value obtained for the Aerial Opt extract was close to the reference LOX inhibitor (quercetin) used in this study, and it could be considered as good [47]. The higher potency of the Aerial Opt extract could have been related to its higher TFC and ROS scavenging capacity, and previous studies have demonstrated and extensively discussed the anti-inflammatory activity (as LOX inhibitors) of flavonoids [48,49]. In addition, these values were similar to those obtained from orange juice by-products and thinned peaches after different drying processes [29,50], which have shown promising neuroprotective potential [51].

### 3.4. Chemical Characterization of Ferula persica L. PLE Extracts

One of the main objectives of the present study was to perform a comprehensive chemical characterization of the *F. persica* aerial part and root extracts. Hence, two complementary untargeted metabolomics approaches were performed: UHPLC-Q-TOF-MS/MS (in ESI (+) and ESI (−) modes) and GC-Q-TOF-MS. Combining the results from all of the extracts (those obtained using H_2_O, EtOH, EtAc, and CPME at 115 °C and those obtained under optimum conditions), 170 and 53 compounds were tentatively identified with high confidence by LC-MS/MS and GC-MS, respectively. It has to be noted that α-humulene was identified in both the LC-MS/MS and GC-MS analyses, but because the confidence in its identification was higher in GC-MS, this compound was only retained in the GC-MS data. In total, 222 compounds belonging to 66 chemical subclasses were identified (Table S2). Among these chemical subclasses, the most represented were “sesquiterpenoids”, “amino acids, peptides, and analogs”, “carbohydrates and carbohydrate conjugates”, “fatty acids and conjugates”, and “alcohols and polyols”, with 27, 19, 15, 15, and 12 compounds, respectively. It is also interesting to note the high number of “hydroxycinnamic acids and derivatives” (such as 2,5-dihydroxycinnamic acid, caffeic acid, 4-caffeoylquinic acid lactone, ferulic acid, 4-coumaric acid, and ethyl caffeate), “benzoic acids and derivatives” (such as benzoic acid, gallic acid, protocatechuic acid, 2-methoxybenzoic acid, 3-formylsalicylic acid, 4-hydroxybenzoic acid, 2,3-dihydroxybenzoic acid, 4-methoxysalicylic acid, and 3,5-dihydroxybenzoic acid), and “flavonoids” (such as apigenin, luteolin, kaempferol, apigenin 7-glucoside, hesperidin, hispidulin 4′-glucoside, isoquercetrin, kaempferol 3-O-(3″,4″-di-O-acetyl-a-L-rhamnopyranoside), luteolin 7-glucoside, luteolin-7,3′-di-O-glucoside, vicenin 2, chrysoeriol, diosmetin, and nobiletin) identified, the presence of which could contribute to the observed TPC and TFC values. In this regard, previous studies conducted on *F. persica* have confirmed the presence of different phenolic compounds and flavonoids (such as quercetin, catechin, vanillic acid, ferulic acid, coumarins, and caffeic acid), some of which have demonstrated neuroprotective activity [44,52,53]. In addition, several compounds that belong to “hydroxycoumarins” (fraxidin, isofraxidin, 7-hydroxy-6-methoxycoumarin, and 6,7-dihydroxycoumarin) and to “quinic acids and derivatives” (quinic acid, chlorogenic acid, neochlorogenic acid, methyl chlorogenate, 5-O-feruloylquinic acid, 3-O-feruloylquinic acid, 3,5-dicaffeoylquinic acid, 4,5-dicaffeoylquinic acid, 1,5-dicaffeoylquinic acid, and 3-O-*p*-coumaroylquinic acid) subclasses were also identified, many of them with reported antioxidant activity [54,55,56]. Previous studies have already reported the chemical composition of *F. persica*, showing sesquiterpene coumarins and sulfur compounds as the main components [57,58]. For instance, 61 components (phenylpropanoids, oxygenated monoterpenes, monoterpene hydrocarbons, sesquiterpenes hydrocarbons, oxygenated monoterpenes, and essential oil) were identified in the aerial parts [57], while 39 compounds (sulfur compounds, oxygenated monoterpenes, and sesquiterpene hydrocarbons) were identified in the roots. In this regard, the present study reports the most comprehensive and exhaustive chemical characterization performed on *F. persica* samples, which is a consequence of combining the use of PLE technology with different solvents, advanced LC/GC-MS approaches, and updated metabolite databases. The abundancy of these compounds widely varied under the different extraction conditions, and the area under the curve of each extraction condition as well as the results of the different statistical analyses are presented in Table S2.

**Figure 1:**
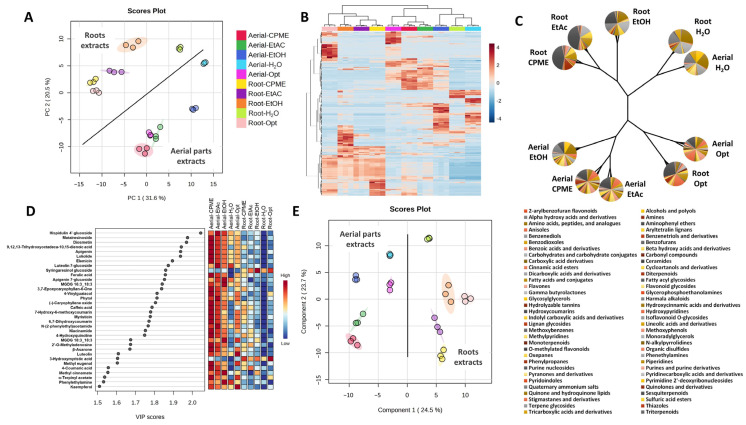
(**A**) PCA score plot of the chemical composition of *Ferula persica* L. aerial part and root PLE extracts; (**B**) Heatmap representation of the chemical composition of *Ferula persica* L. aerial part and root PLE extracts; (**C**) Dendrogram representation of the total abundancy of the different subclasses of compounds present in *Ferula persica* L. aerial part and root PLE extracts (pie plots placed on the ‘leaf’ positions represent the normalized average compound distributions); (**D**) Compounds with VIP scores > 1.5 after PLS-DA analysis; (**E**) PLS-DA score plot of the chemical composition of *Ferula persica* L. aerial part and root PLE extracts.

As a first approach, the overall chemical compositions of the aerial part and root extracts were simultaneously analyzed by PCA, heatmap, dendrogram, and PLS-DA methods (Figure 1). The PCA established two principal components (PC1/PC2) when using the abundancy of the individual compounds, explaining 31.6% (PC1) and 20.5% (PC2) of the variance (Figure 1A). It can be observed that the aerial part extracts were clearly separated from the root extracts, but they shared some similarities: in both matrices, the extracts obtained with EtAc, CPME, and under optimum conditions were very close, while those extracts obtained with EtOH and H_2_O were further separated. The complementary heatmap analysis also clustered together the aerial part extracts obtained with EtAc, CPME, and the optimum conditions (Figure 1B); but, in the case of the roots, the extracts obtained with EtOH and not the optimum conditions were clustered with the EtAc and CPME extracts. In addition, this analysis clustered together the aerial part and root extracts obtained with H_2_O, which were very close to the aerial part extract obtained with EtOH. Moreover, the dendrogram analysis performed using the total abundancy of the different subclasses of compounds provided similar but not exactly the same results (Figure 1C). On the one hand, the EtOH, EtAc, and CPME extracts obtained for the different matrices were grouped together; on the other hand, the aerial part and root extracts obtained with H_2_O and the extracts obtained under optimum conditions were grouped independently. As expected, these results indicated that the different solvents applied to the two matrices affected both the individual compounds and the chemical subclasses to which they belong. Furthermore, the PLS-DA analysis provided 34 compounds with VIP scores > 1.5 (Figure 1D), which were responsible for the separation observed between the different extracts (Figure 1E). This analysis clearly separated the aerial part and root extracts, with hispidulin 4′-glucoside, matairesinoside, diosmetin, 9,12,13-trihydroxyoctadeca-10,15-dienoic acid, apigenin, loliolide, elemicin, luteolin 7-glucoside, syringaresinol glucoside, ferulic acid, apigenin 7-glucoside, and MGDG 16:3_18:3 being the most distinctive variables. Most of these compounds, except syringaresinol glucoside, had higher abundancy in the aerial part extracts, and more specifically in the CPME and EtAc extracts. Other compounds, such as glycoside derivatives (from flavonoids and lignans), had higher abundancy in the EtOH and H_2_O aerial part extracts. Additionally, three interesting compounds that could be pinpointed from this analysis were those with maximum abundancy in the extracts obtained under optimum conditions, such as luteolin and kaempferol (in the aerial part extract) and 3-hydroxymyristic acid (in the root extract). These results demonstrated the different distribution of compounds, such flavonoids, in the different fractions of *F. persica*, but they also highlighted this subclass of compounds, in their free and glycosylated forms, as contributors to the in vitro neuroprotective capacity observed in the extracts obtained under optimum conditions. This hypothesis was also supported by previous studies that have determined that flavonoids, in their free or derivative forms, possess anti-cholinesterase, anti-inflammatory, and antioxidant activities [46,59].

**Figure 2:**
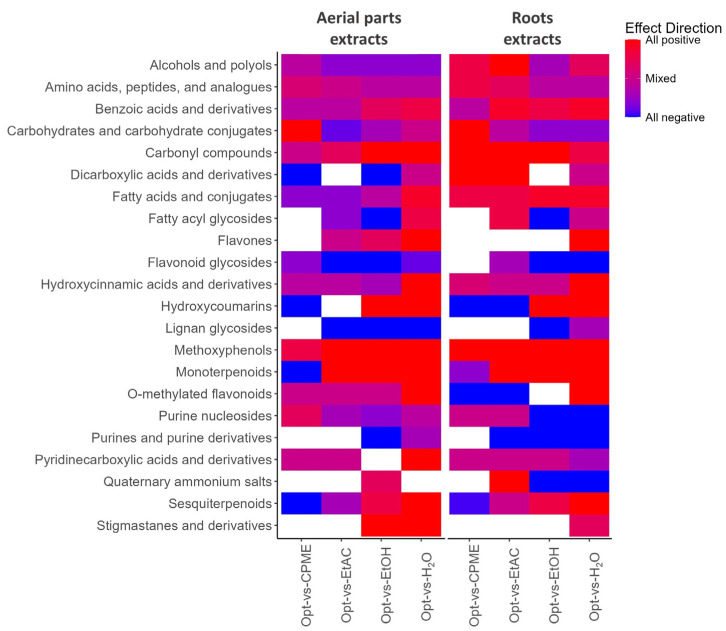
Heatmap representation of the chemical similarity enrichment analysis (based on subclasses of compounds) performed at multiple comparisons (Opt-CPME, Opt-EtAc, Opt-EtOH, and Opt-H_2_O) on the PLE extracts obtained from *Ferula persica* L. aerial parts and roots. Increased clusters (positive) are shown in red while decreased clusters (negative) are colored in blue.

As a second approach, and due to the different chemical composition of the two matrices examined, the aerial part and root extracts were analyzed independently by chemical similarity enrichment analysis (ChemRICH), ANOVA, and Pearson’s correlation. For the ChemRICH analysis, the Simplified Molecular Input Line Entry System (SMILES) and the subclass of each tentatively identified compound were used, as well as the fold change (FC) and *p*-value for each pairwise comparison (Opt-CPME, Opt-EtAc, Opt-EtOH, and Opt-H_2_O) (these values are presented in Table S2). The heatmap obtained from the multiple comparisons showed several chemical subclasses were differently affected by the diverse extraction conditions and matrices used (Figure 2). In the case of the aerial part samples, the optimum conditions highly increased the extraction efficiency of methoxyphenols and monoterpenoids (except with respect to CPME) and other subclasses to a lesser extent (such as amino acids, benzoic acids, carbonyl compounds, flavones, or O-methylated flavonoids). Among the differentially altered methoxyphenols, 4-vinylguaiacol (the decarboxylation product of ferulic acid, formed during thermal treatment) has demonstrated a high antioxidant and anti-inflammatory capacity [60,61], as well as trans-sinapyl alcohol [62], and chrysoeriol (an O-methylated flavonoid) has multiple health-beneficial effects [63]. In the case of the monoterpenoid subclass, five of the identified compounds were acetate derivatives (4-terpinyl acetate, bornyl acetate, fenchyl acetate, lavandulyl acetate, and α-terpinyl acetate), and α-terpinyl acetate and bornyl acetate have demonstrated good neuroprotective potential [64,65]. On the other hand, the extraction of flavonoid glucosides and lignan glucosides was decreased. These last results may have been a consequence of: (i) the use of non-polar solvents (EtAc and CPME), as those compounds are mainly water soluble; and (ii) the use high temperature (180 °C), as this parameter has previously been demonstrated to increase the degradation of glycosides when using PLE technology [66]. In the case of the root samples, the optimum conditions increased the extraction of many subclasses of compounds, such as alcohols and polyols, amino acids, benzoic acids, carbonyl compounds, or methoxyphenols. It is also interesting to point out that hydroxycoumarins, O-methylated flavonoids monoterpenoids, and sesquiterpenoids were more abundant when the extraction was performed with 100% CPME at 115 °C than at optimum conditions (100% CPME at 180 °C), which indicates that temperature plays an important role in their preservation during extraction. In this regard, a number of monoterpenoids and sesquiterpenoids have demonstrated interesting neuroprotective properties [67,68], such as nerolidol, caryophyllene oxide, α-humulene, or α-bisabolol [69,70,71].

**Figure 3:**
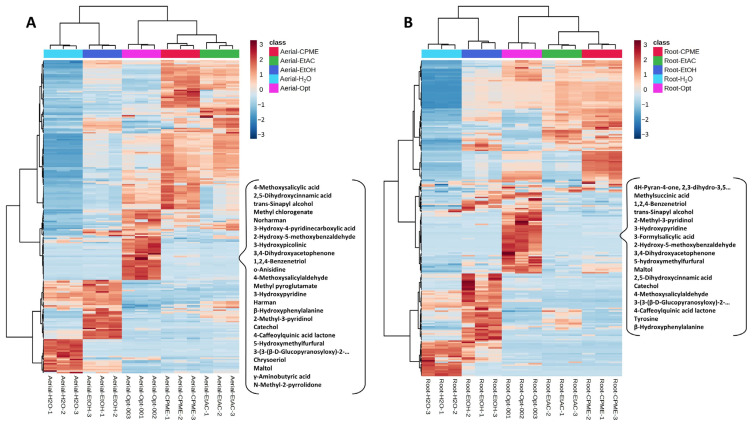
Heatmap representation of the chemical composition of *Ferula persica* L. aerial part (**A**) and root (**B**) PLE extracts, highlighting the compounds mainly present in the extracts obtained under optimum conditions.

Thereafter, the abundancy of the individual compounds was compared among different extracts of the same matrix using ANOVA, revealing that most of them were affected by the extraction conditions (Table S2). A variety of them were mainly present in the extracts obtained under optimum conditions (25 in the aerial part extracts and 18 in the root extracts), as can be observed in the heatmaps in Figure 3. Among these compounds, cinnamic acid derivatives (such as 2,5-dihydroxycinnamic acid) possess the ability to inhibit cholinesterase [72], and β-carbolines (such as harman and norharman) have been described as having good antioxidant, anti-inflammatory, and neuroprotective effects [73]. Other compounds, such as chrysoeriol, can confer neuroprotection [63], and 3,4-dihydroxyacetophenone, which can cross the blood–brain barrier, was shown to be positively correlated with the neuroprotective potential of thinned peaches extracts [50]. Some of these compounds could have been obtained during the extraction process due to the high extraction temperature used (180 °C), which has been shown to help extract phenolic acids [74], releasing free flavonoids from more complex molecules [66] or forming other bioactive compounds, such as melanoidins [75]. This is the case of 5-hydroxymethylfurfural and 4-caffeoylquinic acid lactone, which are known to be formed by thermal processes, such as the thermal decomposition of sugar products or the lactonization of 4-O-caffeoylquinic acid during coffee roasting.

Furthermore, to identity those compounds that could be responsible of the in vitro neuroprotective activity observed, a Pearson’s correlation test between the abundancy of the compounds and ROS and AChE activities was performed (Table S2). In the case of the aerial part samples, this analysis revealed that 50 compounds were significantly correlated with ROS scavenging capacity, while 16 were correlated with AChE inhibitory activity (r < −0.8 and *p*-value < 0.1). Among them, 12 compounds (acetylleucine, 4-methyl-5-thiazoleethanol, β-asarone, *N*-(2-phenylethyl)acetamide, farnesyl acetate, thymidine, cis,cis-linoleic acid, isofraxidin, kaempferol, α-hydroxybutyric acid, nobiletin, and γ-sitosterol) were correlated with both activities (Table 5). In the case of the root samples, and following the same criteria, 38 and 81 compounds were significantly correlated with ROS and AChE activities, respectively. Among them, 8 compounds (guaiol acetate, selina-3,7(11)-diene, 2-hydroxypalmitic acid, kaempferol, stearic acid, palmitic acid, palmitoleic acid, and α-cyperone) were correlated with both activities (Table 5). Some of these compounds, such as isofraxidin [76,77] β-asarone [3], nobiletin [78,79], α-cyperone [70], and kaempferol [43], have already demonstrated interesting neuroprotective properties.

### 3.5. Molecular Docking Simulations

Based on these promising results, molecular docking simulations were performed to evaluate the binding affinities of different complexes between the 19 compounds significantly correlated with ROS and AChE activities and the target enzymes AChE, BChE, and LOX (Table 5). Galantamine and quercetin were used as standard inhibitors. As can be observed, kaempferol (−9.926 kcal/mol), followed by selina-3,7(11)-diene (−9.455 kcal/mol), guaiol acetate (−8.956 kcal/mol), α-cyperone (−8.485 kcal/mol), and farnesyl acetate (−8.015 kcal/mol) had the lowest binding energies for AChE (marked in bold in Table 5), some of them being lower than the complex with the positive control, galantamine (−9.104 kcal/mol). For kaempferol, the low binding energy may have been attributed to the hydrogen bonds formed with the residual amino acids SER125 (the peripheral anionic site) and GLU202 (the anionic subsite) and π–π stacking interactions with amino acids TRP86 (the anionic subsite) and TYR337 (the peripheral binding site) (Figure S1). In the case of selina-3,7(11)-diene, π–σ interactions can occur with the amino acids TRP86 and TYR337 as well as π–alkyl interactions with TYR449, TRP 439, and HIS447 (catalytic triad). And for guaiol acetate, hydrogen bonds formed with the residual amino acids GLY121, GLY122, and SER203 (catalytic triad) and π–σ interactions with TRP439 and π–alkyl interactions with TRP86, TYR449, and TYR337 can occur. Many of these interactions have already been described for compounds targeting acetylcholinesterase [80]. The binding energies of these molecules for BChE enzyme were also low (Figure S2), but only kaempferol (−8.964 kcal/mol) had a lower binding energy than galantamine (−8.646 kcal/mol). However, it must be mentioned that the area under the curve of kaempferol was relatively low (Table S2), which made us discard this molecule as the main molecule responsible of the inhibitory activity observed in vitro. The different binding affinities of these molecules for AChE and BChE could be explained by the different interactions with the catalytic sites of each enzyme, as previously discussed [81]. Finally, it is interesting to note that all of the sesquiterpenes investigated had lower binding energies for LOX than quercetin, the positive control. The lowest binding energy was observed for selina-3,7(11)-diene (−8.685 kcal/mol), followed by guaiol acetate (−8.567 kcal/mol), α-cyperone (−8.124 kcal/mol), and lastly farnesyl acetate (−7.930 kcal/mol). The docking results (Figure S3) showed that most of these compounds had multiple π–alkyl interactions with the same residues: for selina-3,7(11)-diene, with the amino acids LEU565, LEU773, ALA561, HIS523, ILE572, HIS518, VAL769, ILE770, PHE576, and TRP519; for guaiol acetate, with the amino acids LEU565, LEU773, ALA561, HIS523, ILE572, HIS518, HIS513, VAL566, and ILE557 (together with π–σ interactions with TRP519); and for α-cyperone, with the amino acids LEU565, LEU773, ILE572, HIS518, VAL769, PHE576, and LEU 515. Apart from π–alkyl interactions with the amino acids LEU565, LEU773, ALA561, HIS513, ILE572, HIS518, ILE557, ILE770, PHE576, and TRP519, farnesyl acetate also formed hydrogen bonds with ARG726 and GLY720 and π–σ interactions with HIS523. Many of these interactions have already been described for other natural compounds, such as coumarin derivatives and flavonoids [47,82]. Overall, the molecular docking results suggested sesquiterpenes, especially selina-3,7(11)-diene, guaiol acetate, α-cyperone, and farnesyl acetate, as compounds with promising neuroprotective and anti-inflammatory capacity.

## 4. Conclusions

In the present work, a green extraction method based on PLE was optimized to obtain bioactive compounds from *F. persica* L. aerial parts and roots, together with an in vitro neuroprotective evaluation and a comprehensive chemical characterization. The obtained results demonstrate that non-polar solvents in different proportions (EtAc:CPME 79:21% for aerial parts and 100% CPME for roots) and high temperature (180 °C) enhanced the TPC and antioxidant (ORAC assay) results and showed, for the first time, the anti-cholinesterase (AChE and BChE inhibition) and anti-inflammatory (LOX inhibition) capacities of *F. persica* extracts. However, further studies using cell culture or in vivo models are needed to corroborate these activities. Furthermore, the extensive chemical characterization highlighted the complex and diverse composition of all of the extracts, indicating that non-polar solvents increased the extraction of specific subclasses of compounds, such as hydroxycoumarins, flavones, methoxyphenols, monoterpenoids, O-methylated flavonoids, and sesquiterpenoids, independently of the plant material used. Among them, several flavonoids and sesquiterpenoids have already been reported as good antioxidant, anti-inflammatory, and neuroprotective molecules (as those identified in the present work), which combined could explain the in vitro neuroprotective activity observed. Nonetheless, this high complexity also makes the absolute quantification of all molecules difficult and hampers the undoubted determination of the responsible compounds, as synergistic effects might occur. To solve these limitations, future work will be directed to absolutely quantifying the most promising compounds in these extracts, to evaluating their neuroprotective capacity in cell culture models, to investigating their potential to cross the blood–brain barrier, and to performing advanced molecular dynamics simulations if promising results are obtained.

## Figures and Tables

**Table 1:** Extraction yield, TPC, ROS scavenging capacity, and AChE inhibitory activity of PLE extracts obtained using H_2_O, EtOH, EtAc, and CPME, from *Ferula persica* L. aerial parts and roots.

Matrix	Solvent Composition	Extraction Yield (%)	TPC (mg GAE/g)	ROS (IC_50_ μg/mL)	AChE (IC_50_ μg/mL)
Aerial parts	H_2_O	36.7 ± 0.3 ^e^	42.6 ± 0.3 ^b^	2.7 ± 0.1 ^b^	246.1 ± 10.9 ^a,b^
	EtOH	10.9 ± 0.1 ^c^	74.5 ± 0.1 ^c^	1.5 ± 0.2 ^b^	274.9 ± 12.2 ^a^
	EtAc	4.5 ± 0.8 ^a^	76.3 ± 0.2 ^c^	1.1 ± 0.1 ^b^	106.9 ± 6.6 ^d^
	CPME	5.3 ± 0.4 ^a,b^	78.3 ± 0.6 ^c^	1.0 ± 0.1 ^b^	130.2 ± 11.2 ^c,d^
Roots	H_2_O	13.1 ± 0.1 ^d^	20.7 ± 0.3 ^a^	6.3 ± 1.0 ^a^	n.d.
	EtOH	6.2 ± 0.4 ^b^	27.5 ± 0.3 ^a,b^	1.7 ± 0.2 ^b^	n.d.
	EtAc	4.6 ± 0.6 ^a^	19.5 ± 0.3 ^a^	1.6 ± 0.1 ^b^	223.8 ± 16.6 ^a,b,c^
	CPME	4.4 ± 0.1 ^a^	41.6 ± 0.1 ^b^	1.5 ± 0.2 ^b^	173.3 ± 18.4 ^b,c,d^

GAE: Gallic acid equivalents; n.d.: not determined due to the low activity observed. Different letters in the same column indicate significant differences between samples after ANOVA with Tukey’s post hoc test, *p*-value < 0.05.

**Table 2:** Response variables (extraction yield, TPC, ROS scavenging capacity, and AChE inhibitory activity) used to optimize the extraction of bioactive compounds from *Ferula persica* L. aerial parts.

Sample	Temperature (°C)	Solvent Composition	Extraction Yield (%)	TPC (mg GAE/g)	ROS (IC_50_ μg/mL)	AChE (IC_50_ μg/mL)
1	115	50% EtAc:CPME	4.3	64.9 ± 1.7	1.4 ± 0.1	132.8 ± 11.0
2	50	100% CPME	4.2	46.4 ± 0.8	2.1 ± 0.3	205.2 ± 21.3
3	50	50% EtAc:CPME	3.0	64.3 ± 0.2	1.2 ± 0.2	155.1 ± 20.6
4	50	100% EtAc	3.4	58.3 ± 1.1	1.1 ± 0.2	115.3 ± 13.5
5	115	100% CPME	4.9	64.2 ± 0.3	1.5 ± 0.1	167.3 ± 10.9
6	115	50% EtAc:CPME	4.6	68.9 ± 0.6	1.8 ± 0.1	96.9 ± 21.1
7	115	100% EtAc	4.4	67.3 ± 0.6	1.3 ± 0.0	138.3 ± 6.8
8	180	100% CPME	9.3	113.4 ± 0.7	1.1 ± 0.1	109.0 ± 11.3
9	180	50% EtAc:CPME	9.3	116.9 ± 0.5	1.0 ± 0.1	92.2 ± 10.9
10	180	100% EtAc	9.6	120.9 ± 1.0	0.8 ± 0.0	99.9 ± 4.1
11	115	50% EtAc:CPME	6.1	67.8 ± 0.3	1.9 ± 0.2	110.7 ± 16.8

GAE: Gallic acid equivalents.

**Table 3:** Response variables (extraction yield, TPC, ROS scavenging capacity, and AChE inhibitory activity) used to optimize the extraction of bioactive compounds from *Ferula persica* L. roots.

Sample	Temperature (°C)	Solvent Composition	Extraction Yield (%)	TPC (mg GAE/g)	ROS (IC_50_ μg/mL)	AChE (IC_50_ μg/mL)
1	115	50% EtAc:CPME	4.4	22.2 ± 0.4	2.2 ± 0.2	215.2 ± 15.6
2	50	100% CPME	5.0	14.1 ± 0.1	3.1 ± 0.2	345.8 ± 40.1
3	50	50% EtAc:CPME	3.9	19.3 ± 0.5	2.7 ± 0.0	237.4 ± 26.0
4	50	100% EtAc	4.1	15.5 ± 0.2	3.2 ± 0.4	250.8 ± 30.2
5	115	100% CPME	4.8	25.8 ± 0.7	2.4 ± 0.4	193.6 ± 19.0
6	115	50% EtAc:CPME	4.9	22.6 ± 1.1	2.8 ± 0.1	233.9 ± 35.6
7	115	100% EtAc	5.0	14.6 ± 0.7	2.7 ± 0.3	225.4 ± 22.7
8	180	100% CPME	7.0	126.1 ± 2.7	1.3 ± 0.1	116.7 ± 17.2
9	180	50% EtAc:CPME	5.6	62.7 ± 1.1	2.0 ± 0.1	169.8 ± 18.4
10	180	100% EtAc	5.7	54.5 ± 0.6	0.7 ± 0.1	159.9 ± 20.4
11	115	50% EtAc:CPME	4.5	24.0 ± 0.1	1.9 ± 0.2	200.9 ± 25.4

GAE: Gallic acid equivalents.

**Table 4:** Extraction yield, TPC, TFC, and neuroprotective potential evaluation of *Ferula persica* L. extracts (aerial parts and roots) obtained by PLE under optimized conditions.

Sample	Extraction Yield (%)	TPC (mg GAE/g)	TFC (mg QE/g)	ROS (IC_50_ μg/mL)	AChE (IC_50_ μg/mL)	BChE (IC_50_ μg/mL)	LOX (IC_50_ μg/mL)
Aerial Opt	10.1 ± 0.1 *	113.5 ± 3.5	16.0 ± 0.4 *	0.9 ± 0.1	93.6 ± 7.3	142.0 ± 5.0 *	11.6 ± 1.7
Root Opt	7.6 ± 0.5	126.2 ± 3.9 *	3.0 ± 0.2	1.3 ± 0.1 *	133.8 ± 8.0 *	114.9 ± 2.0	23.7 ± 1.9 *
Ascorbic acid				3.7 ± 0.2			
Galantamine					0.4 ± 0.1	3.2 ± 0.3	
Quercetin							11.6 ± 0.7

Aerial Opt: extracts obtained with 79:21% (*v*/*v*) EtAc:CPME at 180 °C; Root Opt: extracts obtained with 100% CPME at 180 °C; GAE: Gallic acid equivalents; QE: Quercetin equivalents. Asterisks indicate significant differences between Aerial Opt vs. Root Opt extracts (for each assay) after two sample *t*-test, *p*-value < 0.05.

**Table 5:** Pearson’s correlation between the relative abundance of different compounds tentatively identified in *Ferula persica* L. extracts (aerial parts and roots) and ROS scavenging capacity and AChE inhibitory activity. Docking score energies of the interactions of the best-docked poses of these compounds in complex with AChE, BChE, and LOX enzymes.

Compound Name	Subclass	Pearson’s Correlation		Binding Energy (kcal/mol)		
		ROS	AChE	AChE	BChE	LOX
Aerial parts						
Acetylleucine	Amino acids, peptides, and analogs	−0.92	−0.93	−6.50	−5.69	−6.02
4-Methyl-5-thiazoleethanol	Thiazoles	−0.85	−0.87	−5.32	−4.82	−5.00
β-Asarone	Anisoles	−0.85	−0.87	−7.33	−5.95	−6.65
*N*-(2-phenylethyl)acetamide	Carboxylic acid derivatives	−0.82	−0.86	−7.73	−6.40	−6.96
**Farnesyl acetate**	**Sesquiterpenoids**	**−0.91**	**−0.85**	**−8.02**	**−6.26**	**−7.93**
Thymidine	Pyrimidine 2′-deoxyribonucleosides	−0.85	−0.85	−7.84	−7.61	−7.293
cis,cis-Linoleic acid	Linoleic acid and derivatives	−0.98	−0.85	−7.26	−6.34	−7.79
Isofraxidin	Hydroxycoumarins	−0.94	−0.84	−7.87	−6.92	−6.79
**Kaempferol**	**Flavones**	**−0.94**	**−0.84**	**−9.93**	**−8.96**	**−7.35**
α-Hydroxybutyric acid	Alpha hydroxy acids and derivatives	−0.94	−0.83	−4.88	−4.52	−4.46
Nobiletin	O-methylated flavonoids	−0.83	−0.82	−5.88	−7.65	−4.72
γ-Sitosterol	Stigmastanes and derivatives	−0.94	−0.81	−4.58	−8.81	−7.01
Roots						
**Guaiol acetate**	**Sesquiterpenoids**	**−0.95**	**−0.99**	**−8.96**	**−8.00**	**−8.57**
**Selina-3,7(11)-diene**	**Sesquiterpenoids**	**−0.97**	**−0.99**	**−9.46**	**−8.22**	**−8.69**
2-Hydroxypalmitic acid	Fatty acids and conjugates	−0.93	−0.99	−6.93	−5.79	−7.02
**Kaempferol**	**Flavones**	**−0.81**	**−0.98**	**−9.93**	**−8.96**	**−7.35**
Stearic acid	Fatty acids and conjugates	−0.91	−0.91	−6.78	−6.34	−7.41
Palmitic acid	Fatty acids and conjugates	−0.96	−0.90	−6.75	−6.00	−7.20
Palmitoleic acid	Fatty acids and conjugates	−0.96	−0.85	−6.69	−6.17	−7.20
**α-Cyperone**	**Sesquiterpenoids**	**−0.87**	**−0.83**	**−8.49**	**−8.09**	**−8.12**
Standard inhibitors						
Galantamine	Galanthamine-type amaryllidaceae alkaloids			−9.10	−8.65	
Quercetin	Flavones					−7.33

## Data Availability

The HPLC-MS/MS and GC-MS data are available within the Supplementary Materials, and raw data can be provided by the corresponding author upon request due to size limitations.

## Supplementary Materials

The following supporting information can be downloaded at: https://www.mdpi.com/article/10.3390/nu16234210/s1. Table S1: Optimum PLE conditions, desirability, and response values predicted by the model and the experimental values obtained from *Ferula persica* L. aerial part and root PLE extracts; Table S2: Tentative identified compounds in *Ferula persica* L. aerial part and root PLE extracts after HPLC-MS/MS and GC-MS analyses; Figure S1: Acetylcholinesterase (AChE) docking complexes with galantamine (commonly known inhibitor) and ligands with the lowest binding energies. The calculated binding energies are shown in red; Figure S2: Butyrylcholinesterase (BChE) docking complexes with galantamine (commonly known inhibitor) and ligands with the lowest binding energies. The calculated binding energies are shown in red; Figure S3: Lipoxygenase (LOX) docking complexes with quercetin (commonly known inhibitor) and ligands with the lowest binding energies. The calculated binding energies are shown in red.
