# Supplementary material for: Optimization and Chemical Characterization of Extracts Obtained from Ferula persica var. latisecta Aerial Parts and Roots and Their Neuroprotective Evaluation
Source: Nutrients. 2024 Dec 5;16(23):4210. doi: 10.3390/nu16234210 (PMC11644649; doi:10.3390/nu16234210)
Supplement: Supplementary file 1 [file nutrients-16-04210-s001.zip › Supplementary Figures.pdf]

Supplementary Figures

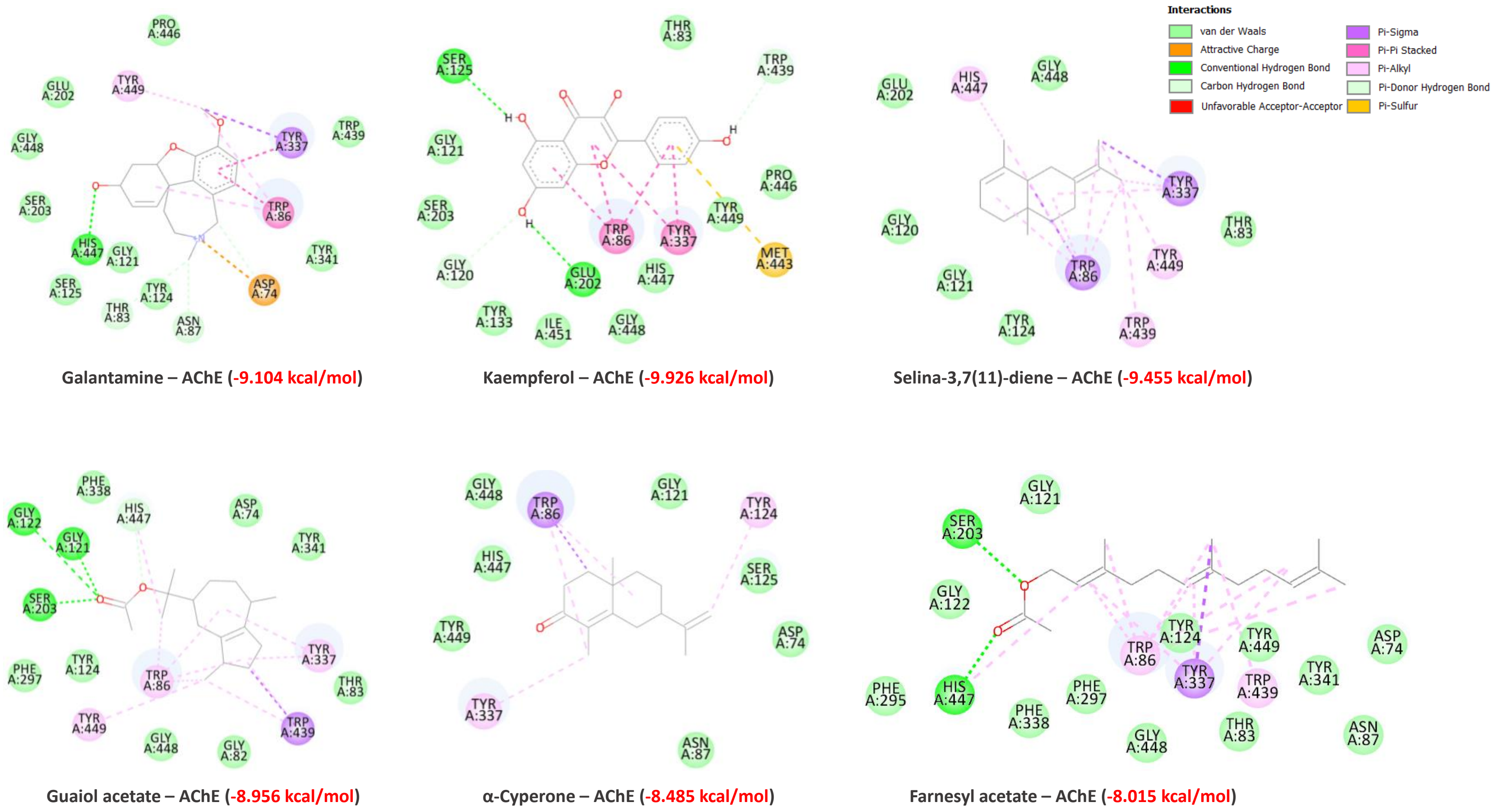

Figure S1. Acetylcholinesterase (AChE) docking complexes with galantamine (commonly known inhibitor) and ligands with the lowest binding energy. In red colour are the calculated binding energies.

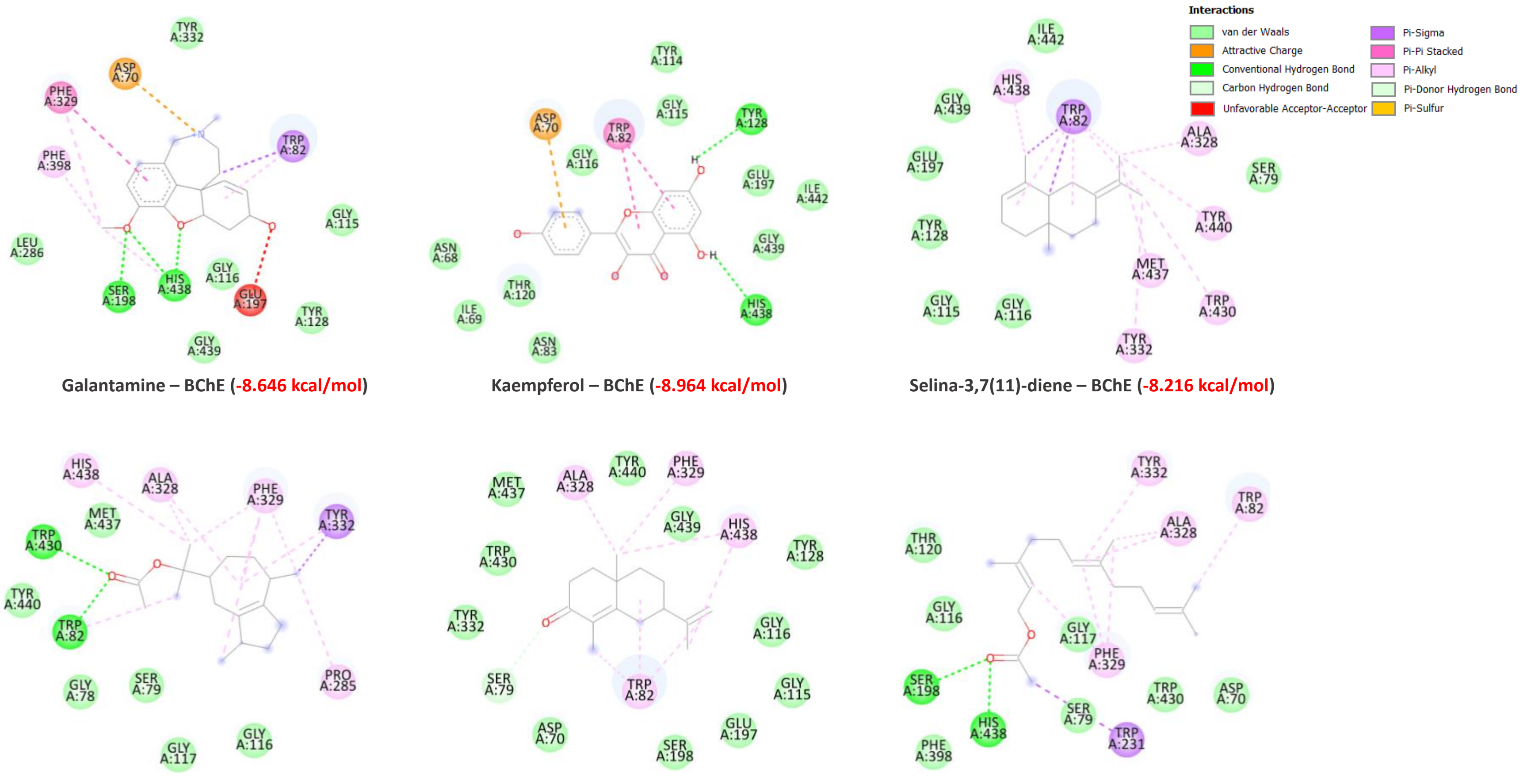

Galantamine – BChE (-8.646 kcal/mol)

Kaempferol – BChE (-8.964 kcal/mol)

Selina-3,7(11)-diene – BChE (-8.216 kcal/mol)

Guaiol acetate – BChE (-8.004 kcal/mol)

α-Cyperone – BChE (-8.093 kcal/mol)

Farnesyl acetate – BChE (-6.260 kcal/mol)

Figure S2. Butyrylcholinesterase (BChE) docking complexes with galantamine (commonly known inhibitor) and ligands with the lowest binding energy. In red colour are the calculated binding energies.

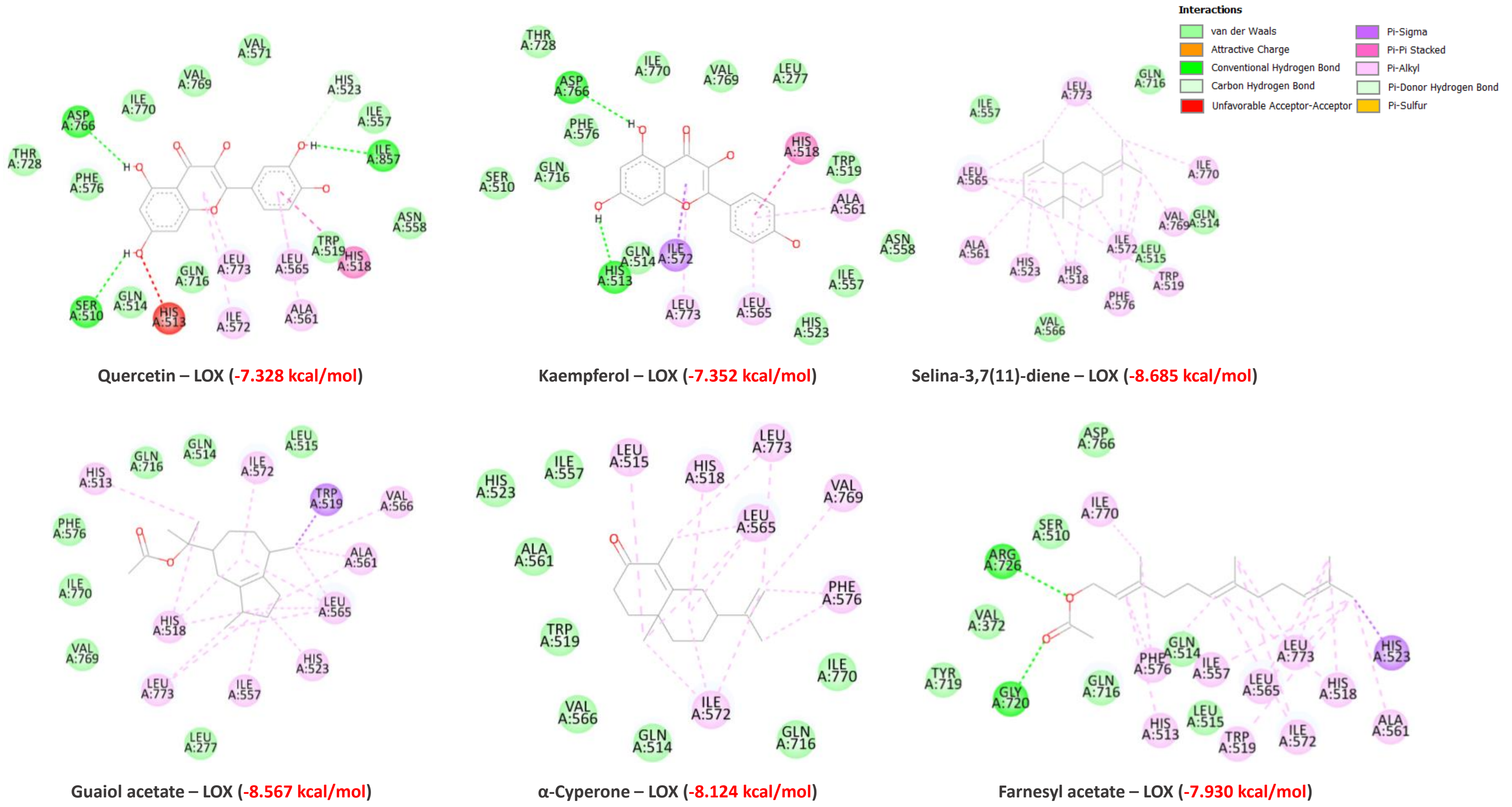

Figure S3. Lipoxygenase (LOX) docking complexes with quercetin (commonly known inhibitor) and ligands with the lowest binding energy. In red colour are the calculated binding energies.
